# Real‐time field‐programmable gate array‐based closed‐loop deep brain stimulation platform targeting cerebellar circuitry rescues motor deficits in a mouse model of cerebellar ataxia

**DOI:** 10.1111/cns.14638

**Published:** 2024-03-15

**Authors:** Gajendra Kumar, Zhanhong Zhou, Zhihua Wang, Kin Ming Kwan, Chung Tin, Chi Him Eddie Ma

**Affiliations:** ^1^ Department of Neuroscience City University of Hong Kong Hong Kong Hong Kong SAR; ^2^ Department of Biomedical Engineering City University of Hong Kong Hong Kong Hong Kong SAR; ^3^ School of Life Sciences, Center for Cell and Developmental Biology and State Key Laboratory of Agrobiotechnology The Chinese University of Hong Kong Hong Kong Hong Kong SAR

**Keywords:** cerebellar ataxia, cerebellum, deep brain stimulation, electroencephalography, electromyography, field‐programmable gate array

## Abstract

**Aims:**

The open‐loop nature of conventional deep brain stimulation (DBS) produces continuous and excessive stimulation to patients which contributes largely to increased prevalence of adverse side effects. Cerebellar ataxia is characterized by abnormal Purkinje cells (PCs) dendritic arborization, loss of PCs and motor coordination, and muscle weakness with no effective treatment. We aim to develop a real‐time field‐programmable gate array (FPGA) prototype targeting the deep cerebellar nuclei (DCN) to close the loop for ataxia using conditional double knockout mice with deletion of PC‐specific LIM homeobox (Lhx)1 and Lhx5, resulting in abnormal dendritic arborization and motor deficits.

**Methods:**

We implanted multielectrode array in the DCN and muscles of ataxia mice. The beneficial effect of open‐loop DCN‐DBS or closed‐loop DCN‐DBS was compared by motor behavioral assessments, electromyography (EMG), and neural activities (neurospike and electroencephalogram) in freely moving mice. FPGA board, which performed complex real‐time computation, was used for closed‐loop DCN‐DBS system.

**Results:**

Closed‐loop DCN‐DBS was triggered only when symptomatic muscle EMG was detected in a real‐time manner, which restored motor activities, electroencephalogram activities and neurospike properties completely in ataxia mice. Closed‐loop DCN‐DBS was more effective than an open‐loop paradigm as it reduced the frequency of DBS.

**Conclusion:**

Our real‐time FPGA‐based DCN‐DBS system could be a potential clinical strategy for alleviating cerebellar ataxia and other movement disorders.

## Graphical abstract

Conventional open‐loop DBS provides continuous stimulation, regardless of changes in physiologic state. Our FPGA‐based closed‐loop DBS system is triggered only when symptomatic muscle EEG is detected in a real‐time manner, which reduced the frequency and time of DBS, preventing over neural stimulation that shortens the battery life of stimulator. The real‐time FPGA‐based closed‐loop DBS system demonstrates a proof‐of‐concept, supporting its potential clinical application in integrating the new generation of implantable pulse generator and external wireless wearable EEG devices for movement disorder.

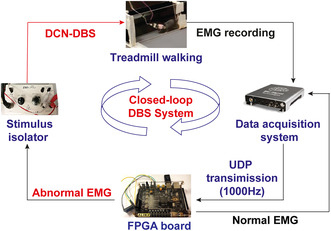

## 1 INTRODUCTION

Deep brain stimulation (DBS) is used widely to improve motor symptoms by exciting afferent and efferent projections.^1^, ^2^, ^3^ Most DBS systems use open‐loop devices that deliver continuous stimulation, regardless of changes in physiologic state. In contrast, closed‐loop DBS is delivered in response to an electrophysiologic biomarker of a disorder detected by either an implanted electrode or wearable sensor. There is substantial evidence showing that excessive current flow to adjacent structure from open‐loop DBS adversely affects motor and cognitive functions.^4^, ^5^, ^6^, ^7^, ^8^ Open‐loop DBS also reduces stimulator battery life, thus increasing the frequency of replacement surgery. It is estimated that >50% of continuously delivered stimulation (i.e., via open‐loop DBS) in patient with Parkinson's disease (PD)—a current therapeutic alternative when medication is inadequate for improving motor symptoms—is unnecessary, and can be avoided by using a feedback biomarker like closed‐loop DBS.^9^

The field‐programmable gate array (FPGA) can be used as a proof‐of‐concept platform to validate methodologies and system architectures, monitor performance, integrate software, debug ports, analyze logic connections, and trace hardware.^10^ FPGA physical interfaces include transistor‐transistor logic input/output, universal serial bus, and ethernet to connect with other systems and extend platform features. FPGA platforms offer a high degree of reusability and optimize system architecture by manipulating buffers, bus bandwidths, and required code space volumes.^11^ Platform performance is monitored to determine real‐time computational operating speed capability. The reconfigurable FPGA integrated circuit uses fixed‐point computation to develop a hardware‐in‐the‐loop real‐time platform and implement bidirectional communications in animal experiments, such as brain‐computer interface (BCI) systems.^12^ BCI acquires physiological signals (e.g., brain signals), analyses them to extract specific features that serves as disease biomarker, and translates them into commands that control application devices like the stimulator for closed‐loop deep brain stimulation.^13^ BCI has been applied across diverse neurotechnological applications, playing a significant role in the development of closed‐loop DBS.

The success of closed‐loop DBS depends on identifying both symptom‐specific biomarkers and selection of an appropriate brain region for DBS. Potential closed‐loop DBS biomarkers have included neural activity like action potentials, electrocorticogram (ECoG), local field potential (LFP), and electroencephalogram (EEG) signals.^14^ Simultaneously sensing and stimulating via the same electrode allows feedback from evoked neural activity. Using peripheral measurements like electromyogram (EMG) signal from symptomatic extremities as the feedback biomarker has been proposed as an application for closed‐loop DBS to treat common movement disorders like PD and essential tremor.^15^, ^16^ Increasing evidence suggests that cerebellar DBS may be an effective therapeutic strategy in movement disorders like nonprogressive ataxia, tremor and dystonia following stroke.^17^, ^18^, ^19^, ^20^, ^21^, ^22^, ^23^ However, closed‐loop DBS is yet in the early stages of development that requires further investigation in searching for what approaches are practical, feasible, and efficient.

In the current study, our goal is to establish online control of the closed‐loop deep cerebellar nuclei (DCN) stimulator in vivo, with stimulation control implemented through a FPGA‐based platform. Closed‐loop DCN‐DBS is triggered only when EMG was detected from symptomatic lower extremities of ataxia mice and analyzed in a real‐time manner, in order to reduce the frequency of DBS. The FPGA system is designed to perform complex computations in real time, and to provide the necessary input/output for simple interfacing in animal experiments. It has high computational speed, completing 1 s real‐world activities within milliseconds,^24^, ^25^, ^26^, ^27^ and generates a stimulus pulse train for DCN stimulation only when needed. Here, we demonstrated application of closed‐loop DBS to treat cerebellar ataxia using EMG signals as an electrophysiologic biomarker with the DCN as the target brain region to close the loop. We used conditional double knockout (DKO) mice with deletion of Purkinje cell (PC)‐specific LIM homeobox (Lhx)1 and Lhx5 in postnatal PCs as a mouse model of cerebellar ataxia. The ataxia (DKO) mice exhibit disrupted cerebellar circuitry leading to nonprogressive cerebellar ataxia (no degeneration of PCs) and severe motor deficits.^28^, ^29^ Our results confirm the superiority of FPGA‐based closed‐loop DCN‐DBS for alleviating cerebellar ataxia. This evidence supports the effectiveness of closed‐loop DCN‐DBS not only for cerebellar ataxia, but also for other movement disorders.

## 2 MATERIALS AND METHODS

### 2.1 Proof‐of‐concept FPGA‐based closed‐loop DBS control system prototype

We designed a closed‐loop DBS control system prototype by integrating hardware and software to automatically trigger stimulation upon detection of an EMG signal below the predefined threshold. The platform integrates a data recording system (Apollo, Bio‐Signal Technologies, USA), FPGA board, and computer, isolated pulse stimulator. The Apollo system collected EMG signals from the GCM of freely moving mice on a treadmill at a constant speed (1‐m/s) (Video S1).

**FIGURE 1:**
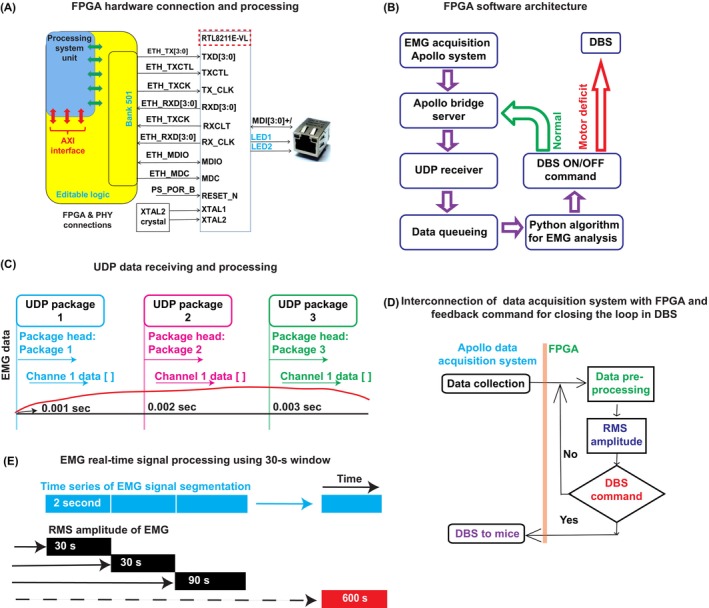
Field‐programmable gate array (FPGA) hardware, software architecture, and data processing for real‐time closed‐loop DBS prototype. (A) Xilinx FPGA board with integrated web server function. Processing unit (blue area) connects interface (red arrow) control to external devise through drive. Features include flexible connectivity of detector front‐end electronics and conventional LINUX‐based computer for higher‐level processing. A cable connects the data acquisition system with the bridge server to the FPGA, and data are transmitted between the FPGA and PHY chip RTL8211E‐VL (red dotted rectangular box) via RGMII bus and transceiver (blue rectangular box). This provides all necessary functions to transmit and receive ethernet packets over CAT 5 UTP cable. (B) FPGA software architecture was designed to execute transmission (Apollo data acquisition to bridge server), receive data (UDP receiver), store data (data queue), process data (EMG threshold detection), and execute decision logics for DBS commands when EMG amplitude is below threshold or execute, command to data recording bridge server to continue data collection and processing. (C) UDP data receiving, and processing was designed to prevent data loss. Three UDP package schematics are shown: and 0.001 s (UDP package 1, blue); 0.002 s (UDP package 2, magenta); 0.003 s (UDP package 3, green). Red line denotes data collection continuity, indicating that 1000 UDP packages are received in 1 s. Thus, the first UPD package will receive all channel data in 0.001 s and the second UPD package will take 0.002 s, denoting no loss of data (i.e., 100% data transmission). (D) Interconnection schematics of data acquisition system with FPGA and feedback command for closing the loop for DBS. FPGA was connected to the data acquisition system and EMG signal was processed in real time; if EMG amplitude was below threshold, DBS was delivered via stimulus isolator; otherwise, a command was sent for data collection and processing. (E) Schematic of EMG real‐time signal processing using 30‐s moving window to improve accuracy and prediction. Optimized 30 s EMG signal epochs were segmented to determine motor function by evaluating the RMS of EMG amplitude. This cycle was repeated for 600 s in one cycle. An automated program was developed to repeat the cycle for 2‐h (which included 1 h DBS and 1 h resting). Commands were projected to a monitor for visualization of the running program.

A FPGA board (ZYNQ7000 AX7020 development board; Xilinx, Beijing, China) with an integrated web server function was used to communicate with the data recording system using a bridge server (developed by Bio‐Signal Technologies, USA) and a cable‐connected computer (cable category 6a, 568B standards) (Figure 1A). A UDP was designed to receive data on the FPGA board, which were saved temporarily in a queue (Figure 1B). The data transfer rate (10 Gb/s) was validated to meet our requirement (140,000 bytes/s) and prevent data loss error (Figure 1C). Real‐time EMG data transfer and on‐line computation of the RMS of EMG amplitude was performed by finalized algorithm tested offline. A Python‐based DBS control program on the FPGA determined the DBS ON/OFF switch status based on the predefined EMG amplitude threshold, then communicated with an isolated pulse stimulator (A‐M System, USA) to deliver a constant current (100 μA) with 130‐Hz biphasic stimulus and 80 μs pulse width to the interposed DCN (Figure 1D).

### 2.2 Experimental animals

All animal experiments were performed in accordance with protocols approved by the Animal Research Ethics Sub‐Committee of the City University of Hong Kong (ref no. A‐0206) and the Department of Health of Hong Kong Special Administrative Region (ref no. 16‐141 in DH/HA&P/8/2/5 Pt.6) and complied with the American Veterinary Medical Association guidelines. The animals were housed in cages on a 12:12‐h light: dark cycle with free access to food and water. Male adult ataxia mice (8–10 weeks old) and age‐matched male littermate mice were used for all experiments. Lhx1/5 DKO mice were generated and maintained on a C57BL6 background as described.^28^, ^29^ Briefly, *PC* protein (*Pcp*)*2*‐Cre mice were crossed with *Lhx1* floxed (fl) and *Lhx5* fl mice to obtain *Pcp2*‐Cre/+; Lhx1^fl/fl^; Lhx5^fl/fl^ DKO mice and Lhx1^fl/fl^; Lhx5^fl/fl^ littermates (control mice). The exact number of animals used in each experiment are described in figure legends. Taking every precaution to minimize the number of animals used. Aside from those with adverse health condition (i.e., drop of implanted electrodes), we did not exclude any animals from analyses.

### 2.3 Behavioral assessments

Investigators who performed behavioral assessments were blinded to mouse genotype. Ataxia and control mouse colonies were maintained by one investigator, and behavioral assessments and DBS were performed by another investigator who was blind to mouse genotype. The investigator who performed EMG and DCN neurospike data analyses was also blinded, via replacement of data file names with codes by a technician who was unaware of mouse genotypes. Mice were trained for 3 sessions on alternate days over 5 days before baseline measurements were collected. Body weight was monitored throughout the study. Improvement of motor deficits in the DCN‐DBS‐treated group was assessed by walking track analysis, pole climbing test (Video S2) and arrow beam walking test (Video S3).^29^, ^30^

#### 2.3.1 Pole climbing test

The pole climbing test is commonly used to assess motor coordination and balance. A wooden pole (50 cm high, 1 cm diameter) was placed vertically on a soft platform. The mouse was gently placed atop the wooden pole with its head up. The time taken to turn around and climb down the pole (i.e., descent latency) was recorded using ANY‐maze software (Stoelting, Kiel, WI, USA) (Video S2). The mouse was allowed to descend the pole repeatedly (including after any fall) during each 2 min training session. During the actual experiment, the mouse was allowed a maximum of 2 min to complete the test. The maximum time (120 s) was recorded for the trial if the mouse fell from the pole or was unable to finish the task. Average motor activity was analyzed and is represented by a heatmap generated using ANY‐maze video tracking software. Mice were tested in 3 independent sessions and averaged values are presented.^29^, ^30^

#### 2.3.2 Narrow beam walking test

Fine motor coordination and balance were assessed using the narrow beam walking test. The apparatus consists of a round wooden beam (100 cm long, 1 cm diameter, placed 50 cm above a table). Mice were trained to cross the beam, which was recorded by a video camera over a period of 2 min using the ANY‐maze automated video tracking system (Video S3). In the event of a fall during training, the mouse was immediately returned to the starting point and allowed another attempt to cross the beam. The maximum time (120 s) was recorded if the mouse fell from the beam during the actual experiment or was unable to finish the task. The time to cross the beam was recorded and is presented as the average of 3 independent trials. Average motor activity is depicted as a heatmap generated using the ANY‐maze video tracking system.^29^, ^30^

#### 2.3.3 Walking track analysis

The walking track analysis test quantifies movement abnormalities in mice. Testing was performed in a quiet room. Mice were acclimatized for 30 min before the test and trained during 3 sessions over 5 days to walk through a narrow corridor covered with white paper (10 × 60 cm). On the day of the actual test, the hind paws and forepaws were painted with red and blue water‐soluble ink, respectively. At least four sets of paw prints were recorded from each mouse and the distance between consecutive right hind paw prints was measured as stride length. The average stride length of each mouse was calculated from 10 to 12 paw prints.^29^, ^30^

### 2.4 GCM EMG recording in freely moving mice

Mice were anesthetized with ketamine (100 mg/kg) and xylazine (10 mg/kg) for customized electrode implantation into the GCM as described previously.^29^, ^31^, ^32^, ^33^ Briefly, a 1‐cm incision on the head was made and the electrode connector was affixed to the skull with dental cement. The wire between the electrode connector and GCM was inserted subcutaneously from the dorsal neck to the leg. Two electrodes 1‐mm apart were implanted into the GCM. The ground wire was implanted in the frontal bone, and the reference wire was inserted between the dura and skull. Extra wire was looped, placed underneath the skin, and sutured. Mice received analgesics, including meloxicam (1 mg/kg) and buprenorphine (75 μg/kg), and they were individually housed for 1 week during recovery.

Spontaneous GCM EMG recording was performed using Apollo II acquisition system (Bio‐Signal Technologies, McKinney, TX, USA), while the mice walked on a treadmill at a constant speed of 1‐m/min. The raw EMG signals were acquired, and the analyses were performed with a custom‐made MATLAB program and Spike2 software (Cambridge Electronic Design, Cambridge, UK). To minimize noise generated by movement artifact, digital high‐pass filter of 50 Hz was used (4th order Butterworth, Bio‐Signal Technologies). The signal was then rectified and smoothed by removing the direct current from the raw EMG signal. RMS of EMG amplitude was detected by setting the minimum threshold to 10% above the rising and falling phases of the EMG signal over the time period of 30 s. RMS of EMG amplitude was calculated by taking square root and average of individual points.^29^, ^34^, ^35^

### 2.5 Multielectrode array (MEA) recording of the interposed nucleus of the DCN

MEA recording was performed using the Apollo II acquisition system (Bio‐Signal Technologies, McKinney, TX, USA) as described.^29^ Briefly, customized 16‐channel MEAs were made by nichrome wire (Gamry Instruments, Warminster, PA, USA) and tested for impedance before use. The electrodes were implanted into the interposed nucleus of the DCN in the right hemisphere at the following coordinates relative to bregma: anteroposterior (AP) = −6.4 mm; mediolateral (ML) = −1.3 mm; and dorsoventral = +2.5–3 mm from the skull surface, and affixed to the skull with dental cement.^29^, ^36^ The reference electrode was placed over the frontal bone. Animal health status was daily monitored for 1 week post‐surgery to ensure no observable differences in motor coordination before and after the electrode implantation. DCN neural spikes were analyzed using Spike2 software (Blackrock Microsystems, Salt Lake City, UT, USA). To avoid electrode drift and background signals, neural spikes were sorted, and principal component analysis was then performed to subtract errors arising from electrode drift. The frequencies of spikes, bursts, and interspike intervals were calculated by a custom‐made MATLAB program.^29^

### 2.6 FPGA‐based closed‐loop DCN DBS

Stimulation and recording electrodes were implanted into the DCN and GCM respectively, after which 1 week of recovery was allowed. DBS parameters for FPGA‐based closed‐loop DCN‐DBS (100 μA current, 130 Hz frequency and 80 μs pulse width) (*n* = 8) were selected based on our previous study in which we tested open‐loop DBS parameters from low (30 Hz) to high (130 or 150 Hz) stimulation frequencies, and systematically varied pulse width values (60 or 80 μs) to maximize motor symptom control in ataxia mice.^29^ Ataxia mice were allowed to walk on a treadmill at a constant speed (1 m/min) for 10‐min sessions with 10‐min breaks between (with no EMG stimulation or recording) to prevent exhaustion.

Closed‐loop DCN‐DBS and EMG recordings were performed for 2 h each day for 7 consecutive days. At the end of day 7, DCN recording only was performed immediately after stimulation. On day 8, after the first stimulation, neurobehavioral tests (walking track analysis, pole climbing, and narrow beam walking tests) were performed to evaluate the efficacy of closed‐loop DCN‐DBS immediately after stimulation. EEG recordings of the cerebellum and motor cortex were obtained after 7 days of DBS in separate batches of animals without performing DCN recordings.

**FIGURE 2:**
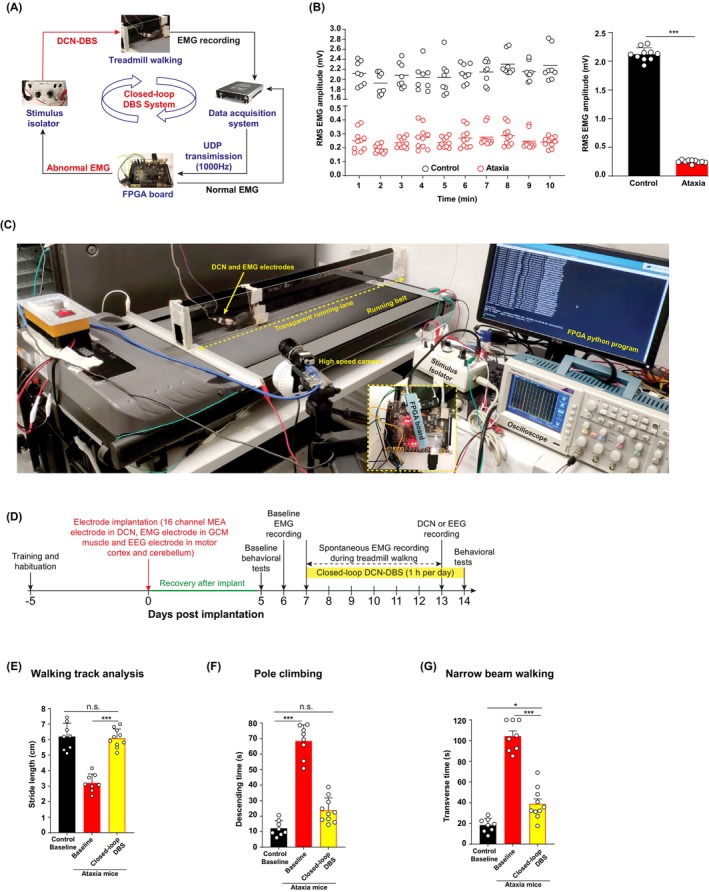
Restoration of motor activity in the ataxia mice via FPGA‐based closed‐loop DCN‐DBS. (A) Schematic of closed‐loop DCN‐DBS paradigm. EMG data were streamed to the FPGA board with a data acquisition system via UDP at 1000 Hz. The RMS of EMG amplitude was analyzed to distinguish abnormal and normal signals. A stimulus pulse train was sent from the FPGA board to the stimulus isolator to trigger DCN stimulation only when symptomatic EMG signal was detected. (B) To determine the average RMS of spontaneous EMG amplitude in the GCM of freely moving mice during treadmill walking at constant speed (1 m/min), spontaneous EMG recording was performed in ataxia and control mice during 10‐min of treadmill walking. Each dot represents average RMS of EMG amplitude per mouse. (C) Mice walked on a treadmill at a constant speed (1 m/min) in a customized transparent running lane (10 cm long, 5 cm wide, to prevent random movements). A tripod‐mounted camera was used to record each trial. Raw EMG signal data were streamed to the FPGA board via a UDP at 1000 Hz, and real‐time data processing was performed to calculate the RMS of EMG amplitude every 30 s. The FPGA board triggered a stimulus pulse train (monitored via oscilloscope) at 130 Hz/80 μs for 30 s only if the RMS value was below a predefined threshold (1 mV). The stimulation pulse train was simultaneously monitored via oscilloscope to ensure successful stimulation delivery. After 30 s of DBS, or if stimulation was determined to be unnecessary, another 30 s of EMG signal was collected and analyzed, and the cycle was repeated. EMG data were saved on the FPGA board for processing with MATLAB. (D) Experimental paradigm for DBS showing training and habituation for 5 days and implantation of multielectrode array (MEA) electrode in the DCN, EMG electrode in the GCM and EEG electrode over the motor cortex and cerebellum. After 5 recovery days, baseline neurobehavioral tests were performed on day 5 and EMG was performed on day 6. Mice were allowed to walk on a treadmill for 10 min followed by a 10‐min break (with no DCN stimulation or EMG recording); this protocol was repeated for a total of 1 h of treadmill walking for 7 days. DCN and EEG recordings were carried out on day 13, immediately after stimulation. Motor behavior was evaluated on day 14, immediately after the last stimulation. (E–G) Performance of ataxia mice returned to baseline (control) levels in the walking track and pole climbing tests after 7 days of closed‐loop DCN‐DBS. The average crossing time for ataxia mice was reduced to near the baseline (control) level after 7 days of DCN‐DBS. Each dot represents average stride length/descending time/traverse time per mouse. Mean ± SEM (*n* = 8–10 mice per group); *p < 0.05, one‐way ANOVA, followed by post hoc Dunnett's multiple comparison test. n.s., not significant (*p* > 0.05).

Closed‐loop DCN‐DBS was controlled with an FPGA board (ZYNQ7000 AX7020 development board; Xilinx, Beijing, China). Prior to the experiment, EMG signal was first observed on the data acquisition system and the EMG channel with the highest signal‐to‐noise ratio was used for signal triggering. For each mouse, we first obtained a baseline EMG signal for 1 min, and then performed online data processing to determine RMS of EMG amplitude. During the 10 min stimulation, 30‐s epochs of raw EMG signal from the selected channel were streamed to the FPGA board via a UDP at 1000 Hz; the board removed the DC from the raw signal. The RMS value of the EMG data was then computed and analyzed onboard. The FPGA board triggered a stimulus pulse train at 130 Hz/80 μs for 30 s only if the RMS value was below the predefined threshold. The pulse train was continuously monitored via oscilloscope to ensure successful delivery (Video S1). After 30 s of DBS, or if stimulation was not delivered, another 30 s of EMG signal were collected and analyzed, and the cycle was repeated. The predefined threshold value was 1‐mV based on the average RMS of EMG amplitude (1.09 ± 0.17 mV) after 7 days of open‐loop DCN‐DBS in ataxia mice.^29^ EMG data were saved on the FPGA board and processed using MATLAB (MathWorks, Natick, MA, USA). The FPGA‐based closed‐loop DCN‐DBS system is shown in Figure 2C and Video S1.

### 2.7 EEG recordings of motor cortex and cerebellum

Mice were anesthetized and epidural stainless steel screw recording electrodes were implanted bilaterally over the parietal cortex (AP = 2 mm and ML = 2 mm relative to bregma) and cerebellum (AP = 6 mm and ML = 2 mm relative to bregma). The reference electrode was implanted over the frontal bone and affixed to the skull with dental cement. Analgesic buprenorphine (75 μg/kg) was administered before and after implantation. Mice were allowed to recover for 5 days post‐surgery. Freely moving EEG signals were recorded using the data acquisition system (Bio‐Signal Technologies). The power spectrum of each spectral band was analyzed with Spike2 and band frequency was determined, as previously described in detail.^37^

### 2.8 Statistical analysis

Data are presented as mean ± standard error of mean (SEM) and were analyzed by repeated measures analysis of variance (ANOVA) with a post hoc Bonferroni test; one‐way ANOVA with a post hoc Dunnett's multiple comparisons test; or two sample Student's *t*‐test, as appropriate and, as stated in figure legends. All graphs were generated and analyzed using Prism v8.0 software (GraphPad, La Jolla, CA, USA).

## 3 RESULTS

### 3.1 Design and optimization of FPGA‐based closed‐loop DBS control system for ataxia

Our FPGA‐based closed‐loop DBS platform was designed to perform real‐time data analysis and deliver DBS current without delay. The FPGA communicated via bridge server with the data acquisition system, whereupon data were transmitted via PHY chip RTL8211E‐VL (red dotted line rectangular box) through the RGMII bus and transceiver (blue highlighted rectangular box) (Figure 1A). The FPGA platform transmitted and received ethernet packets over a CAT 5 UTP cable.

The goal was to develop a “DCN stimulator” capable of delivering patterned neural spike trains to the DCN, to normalize motor coordination and balance deficits in ataxia mice from cerebellar malfunction. To achieve this, we integrated EMG activity and DCN electrophysiology activity to create a hardware prototype system (i.e., FPGA) for real‐time spike generation to test in ataxia mice. The FPGA board was based on a matrix of configurable logic blocks which are customizable to desired applications and functionality requirements. EMG data were transferred in real‐time using the bridge server, which received the unpacked data. A User Datagram Protocol (UDP) was designed to receive data on the FPGA board using the UDP receiver and saved temporarily as Redish (share data) queuing (Figure 1B). Next, we checked for loss of data during transmission by UDP. A schematic of three UDP packages of EMG channels are shown at: 0.001 s (UDP package 1, blue), 0.002 s (UDP package 2, magenta); and 0.003 s (UDP package 3, green), with red line denoting continuity of data collection. The 1000 UDP packages were transmitted and received in 1 s, suggesting no loss of data (i.e., 100% data transmission) (Figure 1C). We subjected the interconnected data acquisition system with FPGA and feedback command to close the loop via DBS (Figure 1D). EMG data collection and root mean square (RMS) amplitude calculation were performed every 30‐s in real‐time for 10‐min using a moving window method (Figure 1E).

To establish the FPGA‐based closed‐loop DCN‐DBS prototype, we streamed EMG data obtained using a UDP at 1000 Hz to the FPGA board. The root mean square (RMS) of EMG amplitude was analyzed on the board to distinguish symptomatic (i.e., below the predefined EMG threshold) from normal EMG signals. A stimulation pulse train was sent from the FPGA board to the stimulus isolator to stimulate the DCN only when the RMS EMG amplitude was below the predefined threshold, which was simultaneously monitored via oscilloscope to ensure successful delivery (Figure 2A). To achieve this goal, we used ataxia mice with PCs characteristically exhibited thinner dendrites and abnormal dendritic spine morphology.^28^ Lhx1 and Lhx 5 are LIM homeodomain transcription factors, which are essential for early PC differentiation and maturation.^28^, ^38^, ^39^, ^40^ The ataxia mice exhibited severe motor deficits.^28^, ^29^ To enable detection of EMG activities in freely moving animals, we first defined EMG threshold based on the average RMS of spontaneous EMG amplitude in the gastrocnemius muscle (GCM) of freely moving mice during treadmill walking at a constant speed (1 m/min). The average RMS of spontaneous EMG amplitude in control and ataxia mice were 2.12 ± 0.03 and 0.25 ± 0.01 mV, respectively (Figure 2B). Our recent study on open‐loop DCN‐DBS showed that the average RMS EMG amplitude of ataxia mice was 1.09 ± 0.17 mV after 7 days of open‐loop DCN‐DBS, in which the ataxia mice regained most of the motor function.^29^ We therefore set the pre‐defined threshold RMS EMG amplitude value as 1 mV. DCN‐DBS was triggered only when the EMG amplitude was below the EMG threshold of 1 mV, which was at least 50% of the control baseline value (2.12 mV)^41^ and was significantly higher than the average baseline RMS EMG value for ataxia mice (0.25 mV) (Figure 2B).

To perform closed‐loop DCN‐DBS in freely moving mice, stimulation and recording electrodes were implanted into the DCN and GCM, respectively, and the mice walked on a treadmill at a constant speed (1 m/min) for 10 min followed by a 10‐min break during which there was no DCN stimulation or EMG recording to prevent exhaustion. Cumulative treadmill walking was 1 h. Prior to DBS, the EMG signal was observed on the data acquisition system and the channel with the highest signal‐to‐noise was selected to control the DBS. For each animal, the FPGA board first collected baseline EMG signal for 1‐min, and online data processing was performed to determine the baseline RMS of EMG amplitude. During DBS, each 30‐s epoch of raw EMG signal from the selected channel was collected on the FPGA board, after which the RMS values of these data were computed on‐board in real time. The FPGA board triggered for 30 s only if the RMS of EMG amplitude was below the predefined threshold value. After 30 s of DBS, or if stimulation was deemed unnecessary, another 30 s of EMG signal were analyzed, and the cycle was repeated (Figure 2C and Video S1).

### 3.2 FPGA‐based closed‐loop DCN‐DBS to restore motor function and EEG oscillations in ataxia mice

To evaluate the therapeutic potential of our FPGA‐based closed‐loop DCN‐DBS prototype for treating cerebellar ataxia, ataxia and control mice received 1 h of daily DCN stimulation for 7 consecutive days and underwent a series of spontaneous motor function tests (Figure 2D). The selected DBS parameters (100 μA/130 Hz/80 μs) was based on our recent study on open‐loop DCN‐DBS,^29^ and was also commonly used for cerebellar DBS in patients and animal models of movement disorders such as tremor, cortical ischemic stroke, and dystonia.^21^, ^23^, ^36^ To examine the motor coordination of ataxia mice after 7 days of closed‐loop DCN‐DBS, we performed walking track analysis, pole climbing test, and narrow beam walking test. Control mice were also assessed. Overall motor performance of ataxia mice receiving closed‐loop DCN‐DBS was significantly higher than that of ataxia mice. Gait performance (walking track analysis) (Figure 2E) and motor ability (pole climbing) (Figure 2F) in ataxia mice returned to baseline levels after 7 days of closed‐loop DCN‐DBS (Video S2). By presenting the highly challenging motor task last, ataxia mice (104.2 ± 3.8 s) took much longer time to cross the narrow beam compared with control littermates (18.3 ± 3.8 s); however, the average crossing time for ataxia mice decreased to 38.9 ± 3.3 s after 7 days of DCN‐DBS (Figure 2G and Video S3).

**FIGURE 3:**
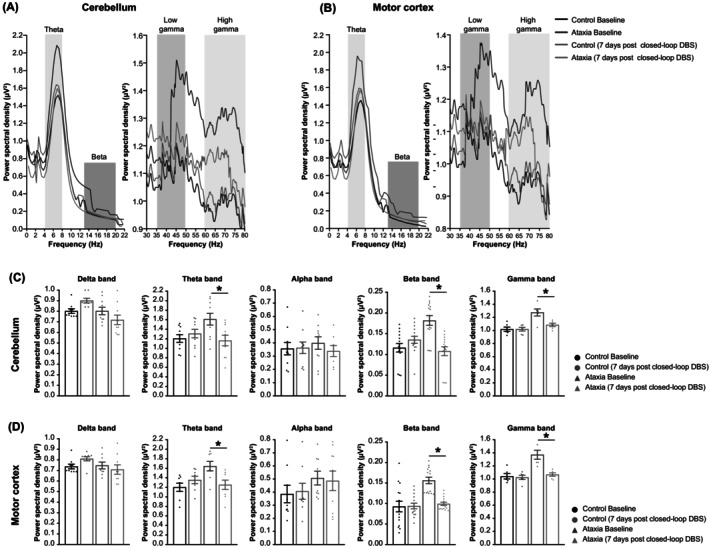
Restoration of motor cortex and cerebellar EEG oscillations in ataxia mice via closed‐loop DCN‐DBS. Oscillatory activity in the cerebello‐cortical network was analyzed by simultaneous EEG recordings of the (A) cerebellum and (B) motor cortex in awake, active mice. Average power spectral densities, where *X*‐axis is frequency (Hz); *Y*‐axis is power spectral density (μV^2^ of each frequency). Significant increases in theta (4–8 Hz), beta (13–20 Hz), and gamma (30–80 Hz) power were detected in the (C) cerebellum and (D) motor cortex of ataxia mice. Each dot represents average power spectral density per mouse. Mean ± SEM (control, *n* = 8; ataxia, *n* = 10); *p < 0.05, one‐way ANOVA, followed by post hoc Bonferroni's test.

Electroencephalogram and neurospike analyses further demonstrated successful development of a FPGA‐based closed‐loop DCN‐DBS system. We examined oscillatory activity in the cerebello‐cortical network by simultaneous cerebellar and motor cortex EEG recordings in awake, active mice. These EEG spectra were computed over 0–80 Hz frequency range at a resolution of 0.25 Hz (Figure 3A,B). Significant increases in theta (4–8 Hz), beta (13–20 Hz), and gamma (30–80 Hz) powers in both brain areas were detected in ataxia mice, while delta (0–4 Hz) and alpha (8–13 Hz) powers were unaffected in ataxia mice with or without DBS (Figure 3C,D). These data indicate that closed‐loop DCN‐DBS reduced movement‐related EEG oscillatory activity and restored a baseline state cerebello‐cortical network.

### 3.3 Closed‐loop paradigm increases the efficacy and energy efficiency in DCN‐DBS

**FIGURE 4:**
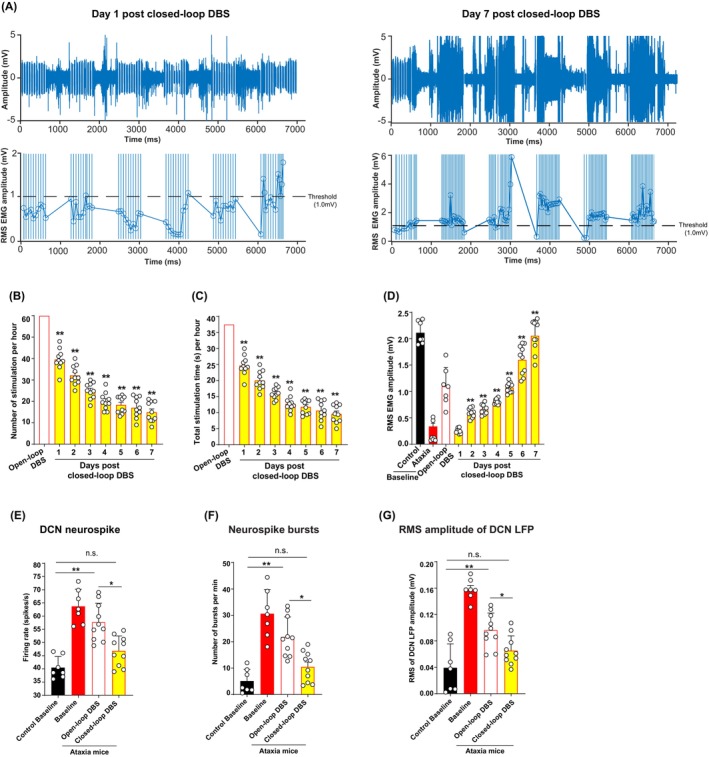
Closed‐loop paradigm increases efficacy and energy efficiency of DCN‐DBS. (A) Representative raw EMG trace (upper panel). Average RMS of EMG amplitude was markedly increased after 7 days of closed‐loop DCN‐DBS in ataxia mice (lower panel) (*n* = 7–10 mice per group). The predefined threshold of RMS of EMG amplitude was 1 mV. Open circles indicate time points when EMG activity was recorded. (B) Closed‐loop DCN‐DBS reduced total stimulations by 75%, compared with open‐loop DCN‐DBS. Each dot represents number of stimulations per mouse. Mean ± SEM (*n* = 7–10 mice per group); **p < 0.001, two‐way ANOVA for repeated measures, followed by post hoc Bonferroni test. (C) Closed‐loop DCN‐DBS reduced the total amount of stimulation time by 74% across 7 days DBS. Each dot represents total stimulation time per mouse. Mean ± SEM (*n* = 7–10 mice per group); **p < 0.001, two‐way ANOVA for repeated measures, followed by post hoc Bonferroni test. (D) Closed‐loop DCN‐DBS restored RMS of EMG amplitude to baseline (control) level after 7 days of stimulation. Each dot represents average RMS of EMG amplitude per mouse. Mean ± SEM (*n* = 7–10 mice per group); **p < 0.001, two‐way ANOVA for repeated measures, followed by post hoc Bonferroni test. (E–G) Total neural spikes, number of bursts, and RMS of EMG amplitude of DCN LFP in ataxia mice were all restored to nearly baseline levels after closed‐loop DBS. Each dot represents average firing rate/number of bursts/RMS of DCN LFP amplitude per mouse. Mean ± SEM (*n* = 6–8 mice per group); *p < 0.05, **p < 0.001, one‐way ANOVA, followed by post hoc Dunnett's multiple comparison test. n.s., not significant (*p* > 0.05).

To evaluate the effectiveness of closed‐loop DCN‐DBS, we compared the average number of stimulations triggered by the open‐loop versus closed‐loop paradigm. Based on the closed‐loop DBS paradigm (30‐s RMS of EMG recording and 30 s stimulation within 1 min, Figure 1E), open‐loop DBS can trigger 60 stimulations (1 stimulation per min) during a 1‐h DBS session, while closed‐loop DBS triggered only 15.42 stimulations/hour on day 7 post DBS (Figure 4A,B). Closed‐loop DBS greatly reduced the total amount of stimulation time by 74%, when compared total open‐loop DBS time 37.44 s/h (100 μA/130 Hz/80 μs: 130 × 80 μs × 60 s × 60 stimulations) with total closed‐loop DBS time 9.62 s/h (100 μA/130 Hz/80 μs: 130 × 80 μs × 60 s × 15.42 stimulations) in 7 days (Figure 4C). Closed‐loop DCN‐DBS completely restored RMS EMG amplitude to the baseline level after 7 days of DBS, whereas open‐loop DBS restored the amplitude by 60% of control baseline values (Figure 4D).^29^ We further compared the 2 paradigms in terms of symptom suppression efficiency by electrophysiologic recordings. In vivo multielectrode array (MEA) recordings revealed that DCN neural firing in ataxia mice in the closed‐loop DCN‐DBS group was close to the baseline levels after 7 days of DBS. Total neural spikes, number of bursts, and RMS amplitude of DCN LFP all returned to nearly baseline levels in freely moving mice that received less amount of stimulation in closed‐loop DCN‐DBS (Figure 4E–G).

## 4 DISCUSSION

Deep brain stimulation is a standard of care for patients with movement disorders like PD, essential tremor, and dystonia, and is an effective treatment for those who become unresponsive to conventional pharmacologic treatments^42^ or have a refractory neurological disorder.^43^, ^44^, ^45^ However, standard, preprogrammed DBS and stimulation parameters do not automatically respond to the patient's needs in real time. The use of constant stimulation provides stimulation in excess of that which is warranted by symptoms can cause adverse side effects. To address these issues, use of a FPGA‐based platform in BCI devices and closed‐loop controls have been reported.^46^, ^47^, ^48^ Recently, closed‐loop DBS for PD treatment has shown promising clinical study results, with reduced side effects and lower‐power consumption compared with conventional open‐loop DBS.^49^ Therefore, implementing FPGA‐based platforms may hold vital significance for future development of microchip‐based devices. Here, we successfully developed a FPGA‐based closed‐loop DCN‐DBS prototype using RMS of EMG amplitude as an electrophysiologic biomarker, so that DBS is triggered only when stimulation is required to close the loop, in response to ongoing movement and symptom intensity. The threshold RMS of EMG amplitude for triggering DBS was automatically and dynamically adjusted based on a symptomatic EMG signal, preventing neuronal overstimulation. Closed‐loop DCN‐DBS greatly reduced the amount of stimulation required to restore motor activity in ataxia mice and was more effective than an open‐loop paradigm in restoring neuronal activity.

Electromyography recording allows detection of functional neuromuscular synapses at target muscles and is widely used to quantify motor function recovery^29^, ^31^, ^34^, ^50^, ^51^, ^52^ and motor deficits in movement disorders. For instance, surface EMG (sEMG) from symptomatic extremities can be used to predict the onset of tremor in essential tremor patient^53^ with an accuracy of over 80% in PD and essential tremor patients.^15^ DBS was triggered only when the EMG activity of the deltoid muscle in the upper extremity of essential tremor patient decreased below the threshold.^54^ EMG movement‐triggering closed‐loop DBS system has been used to close the loop in essential tremor and performed better than the tremor‐modulated closed‐loop DBS system even though the EMG movement‐triggering system will still trigger stimulation when the patients make movement that do not result in tremor.^55^ Motor function depends on the integrity of descending motor pathways that ultimately activate target muscles.^31^, ^56^ We recently reported that disruption of cerebellothalamocortical motor circuitry in ataxia mice significantly delays EMG onset and reduces EMG amplitude after electrical or optogenetic DCN stimulation, suggesting an alternation of cerebellar outputs due to the disruption of spinal motor circuits, resulting in reduced motor activity.^31^ Several clinical reports suggest that chronic muscle denervation is a sign of PD, for which EMG tests play a critical diagnostic role.^57^, ^58^ Although beta power in the subthalamic nucleus (STN) is a useful metric in PD, beta oscillations, while linked to clinical symptoms, are modulated by external factors like medication, movement, speaking, and cognition. Stimulation can cause artifacts when the recording is performed nearby, despite the fact that this can be avoided by ECoG sensing. However, it is nonetheless an invasive method, involving subdural electrodes placements. Prolonged implantation and anesthesia can exacerbate brain injury. Therefore, EMG recordings or kinematic sensors would be considered as more attractive feedback biomarkers for closed‐loop DBS for cerebellar ataxia and movement disorder treatments.

Our electrophysiology studies showed that the cerebello‐cortical network of ataxia mice was restored to baseline after 7 days of closed‐loop DBS. This result suggests that rather than simply modulating the activity of individual neurons, DBS remodels entire neural circuits, which requires further investigation. We also demonstrated that closed‐loop DBS led to restoration of normal cerebellar and motor cortex EEG spectra within the theta, beta, and gamma frequencies. Notably, beta activity amplitude is positively correlated with motor symptom severity,^59^ while reductions in rigidity and bradykinesia have been linked to decrease in beta activity.^60^ Consistent with this, beta frequency oscillatory firing in the STN is suppressed by dopaminergic drugs, with corresponding clinical improvements in akinesia, bradykinesia, rigidity, and tremor.^61^ In contrast, dyskinesia symptoms have been associated with increases in theta and gamma frequencies.^62^ With the recent advance in new generation of implantable pulse generator (IPG), which allows bi‐directional neural interfaces to support sensing and stimulation simultaneously (also known as adaptive DBS).^63^, ^64^ In a recent study, beta band activity has been used as a biomarker for motor improvement in PD patients with bradykinesia using the Percept IPG.^65^ Given the heterogeneity of clinical symptoms, a combination of feedback signals and neural biomarkers are likely to be superior to any single measure for closing the loop in DBS.

In summary, our FPGA‐based closed‐loop DBS system demonstrates a proof‐of‐concept, supporting its potential clinical application in integrating existing deep brain neurostimulators and external wearable EEG devices. However, our FPGA‐based closed‐loop DBS system have some limitations: that is, it might be inconvenient for clinical use since additional hardware/battery for measuring EMG must be implanted and the entire system is connected with wires running most of the length of the body which is susceptible to breakage. Further, there is only one muscle from the upper or lower extremity can be sensed from a single sensor. We expect the development of low‐power wearable wireless EMG sensor with low battery consumption since wireless transmission shortens the life of battery. Nevertheless, the exterior battery in the sensor can be easily replaced that do not require any surgical intervention. Next‐generation closed‐loop or adaptive DBS will likely have even greater flexibility to respond to multiple feedback signals and peripheral electrophysiologic biomarkers, with sophisticated algorithms adjusting simulation parameters for maximal motor function recovery. This scenario is feasible given that it is currently possible to perform simultaneous electrophysiological recordings and stimulation using the same electrode in humans.^66^ Additionally, ataxias are a heterogenous group of movement disorders with a variety of underlying causes in which the high‐frequency stimulation parameters and the use of EMG as biomarker may not have high relevance across different types of ataxias. In fact, a number of studies demonstrate that cerebellar DBS at low frequency (30 Hz or lower) effectively improves degenerative ataxia.^67^, ^68^, ^69^, ^70^, ^71^ In the current study, the PCs of ataxic mice remain intact with abnormal dendritic arborization, suggesting that the ataxic phenotype is not preliminary caused by the degeneration and loss of PCs. Our recent study demonstrated that intact PCs with reduced dendritic arborization are more responsive to a broader range of open‐loop DCN‐DBS stimulation paradigms.^29^ The effectiveness of cerebellar DBS relies heavily on PC neurotransmission given that ataxia mice with genetically eliminating PC GABAergic neurotransmission resulting in no improvements after cerebellar DBS.^70^ Therefore, the current study paves the way for future development of bidirectional neural prostheses for the treatment of ataxia as well as other movement disorders. Recent clinical studies of cerebellar DBS for severe dystonia^72^ and Phase I clinical trial for post‐stroke motor rehabilitation^73^ highlight the use of cerebellar DBS as a potential intervention in various movement disorders.

## AUTHOR CONTRIBUTIONS

G.K. performed the surgery, in vivo animal behavioral assessments, in vivo recordings and electrophysiology data analysis. Z.Z. standardized all computer programs for FPGA board analysis and closed‐loop strategy. Z.W. optimized EMG recording in freely moving mice. K.M.K. provided the Lhx1/5 DKO mice and genotyping protocol. C.T. established and standardized computer programs for FPGA board analysis. C.H.E.M. conceived the project, designed the study and wrote the manuscript with inputs from all authors. All authors read and approved the manuscript.

## CONFLICT OF INTEREST STATEMENT

The authors declare no competing financial interests.

## Supporting information


Video S1.

Video S2.

Video S3.

## Data Availability

The data that support the findings of this study are available from the corresponding author upon reasonable request.
