# Supplementary material for: Real‐time field‐programmable gate array‐based closed‐loop deep brain stimulation platform targeting cerebellar circuitry rescues motor deficits in a mouse model of cerebellar ataxia
Source: CNS Neurosci Ther. 2024 Mar 15;30(3):e14638. doi: 10.1111/cns.14638 (PMC10941591; doi:10.1111/cns.14638)
Supplement: Supplementary file 1 — Video S1. [file CNS-30-e14638-s003.zip › VideoS1Caption.docx]

**Video** **S1.** FPGA-based closed-loop DBS system. Top, DBS was not triggered when a mouse with normal EMG signal walked on the treadmill. The monitor on the right shows the continuous EMG recording. The mouse without DBS had difficulty walking on the treadmill. Bottom, DBS was triggered (beginning of video) when a mouse with an abnormal EMG walked on the treadmill. The stimulus pulse train was observed via oscilloscope during the recording to confirm successful stimulus delivery. The monitor on the right shows continuous EMG recording at the end of the video, with the cycle repeating. The mouse with DBS did not show any difficulty in walking.
