# Supplementary material for: Real‐time field‐programmable gate array‐based closed‐loop deep brain stimulation platform targeting cerebellar circuitry rescues motor deficits in a mouse model of cerebellar ataxia
Source: CNS Neurosci Ther. 2024 Mar 15;30(3):e14638. doi: 10.1111/cns.14638 (PMC10941591; doi:10.1111/cns.14638)
Supplement: Supplementary file 2 — Video S2. [file CNS-30-e14638-s002.zip › VideoS2Caption.docx]

**Video** **S2.** Pole-climbing performance of ataxia mice after 7 days of closed-loop DCN-DBS (or without DBS) using optimal DBS parameters (100 μA/130 Hz/80 μs). Left, ataxia mouse without DBS remained immobile and stayed at the top of the pole for most of the trial. Right, ataxia mouse with DBS descended the pole with ease.
