# Supplementary material for: Real‐time field‐programmable gate array‐based closed‐loop deep brain stimulation platform targeting cerebellar circuitry rescues motor deficits in a mouse model of cerebellar ataxia
Source: CNS Neurosci Ther. 2024 Mar 15;30(3):e14638. doi: 10.1111/cns.14638 (PMC10941591; doi:10.1111/cns.14638)
Supplement: Supplementary file 3 — Video S3. [file CNS-30-e14638-s001.zip › VideoS3Caption.docx]

**Video** **S3.** Narrow beam walking performance of ataxia mice after 7 days of closed-loop DCN-DBS using optimal DBS parameters (100 μA/130 Hz/80 μs). Top, ataxia mouse without DBS remained immobile and stayed at the starting point for most of the trial. Bottom, ataxia mouse with DBS crossed the beam with ease.
